# Metformin acts as a dual glucose regulator in mouse brain

**DOI:** 10.3389/fphar.2023.1108660

**Published:** 2023-04-20

**Authors:** Bo-Yeong Jin, Hyun-Ju Kim, Mi-Jeong Oh, Na-Hee Ha, Yong Taek Jeong, Sang-Hyun Choi, Jun-Seok Lee, Nam Hoon Kim, Dong-Hoon Kim

**Affiliations:** ^1^ Department of Pharmacology, Korea University College of Medicine, Seoul, Republic of Korea; ^2^ Graduate School of Medicine, Korea University, Seoul, Republic of Korea; ^3^ Division of Endocrinology and Metabolism, Department of Internal Medicine, Korea University College of Medicine, Seoul, Republic of Korea

**Keywords:** metformin, hypothalamus, gut-brain axis, glucose regulation, gastric emptying, hepatic gluconeogenesis

## Abstract

**Aims:** Metformin improves glucose regulation through various mechanisms in the periphery. Our previous study revealed that oral intake of metformin activates several brain regions, including the hypothalamus, and directly activates hypothalamic S6 kinase in mice. In this study, we aimed to identify the direct effects of metformin on glucose regulation in the brain.

**Materials and methods:** We investigated the role of metformin in peripheral glucose regulation by directly administering metformin intracerebroventricularly in mice. The effect of centrally administered metformin (central metformin) on peripheral glucose regulation was evaluated by oral or intraperitoneal glucose, insulin, and pyruvate tolerance tests. Hepatic gluconeogenesis and gastric emptying were assessed to elucidate the underlying mechanisms. Liver-specific and systemic sympathetic denervation were performed.

**Results:** Central metformin improved the glycemic response to oral glucose load in mice compared to that in the control group, and worsened the response to intraperitoneal glucose load, indicating its dual role in peripheral glucose regulation. It lowered the ability of insulin to decrease serum glucose levels and worsened the glycemic response to pyruvate load relative to the control group. Furthermore, it increased the expression of hepatic *G6pc* and decreased the phosphorylation of STAT3, suggesting that central metformin increased hepatic glucose production. The effect was mediated by sympathetic nervous system activation. In contrast, it induced a significant delay in gastric emptying in mice, suggesting its potent role in suppressing intestinal glucose absorption.

**Conclusion:** Central metformin improves glucose tolerance by delaying gastric emptying through the brain-gut axis, but at the same time worsens it by increasing hepatic glucose production via the brain-liver axis. However, with its ordinary intake, central metformin may effectively enhance its glucose-lowering effect through the brain-gut axis, which could surpass its effect on glucose regulation via the brain-liver axis.

## 1 Introduction

Metformin is one of the most widely prescribed anti-diabetic drugs for type 2 diabetes mellitus. Many studies have proposed its mechanisms underlying its mode of action in the periphery; these include a decrease in hepatic glucose production, inhibition of intestinal glucose absorption, and improvement of insulin sensitivity, which are presumably mediated via the inhibition of mitochondrial respiratory chain complex 1, activation of adenosine monophosphate-activated protein kinase (AMPK), enhancement of incretin axis, and counter-regulatory action on glucagon (Foretz et al., 2014; Thomas and Gregg, 2017). However, a direct role of metformin via the brain, in glucose metabolism, remains unclear, given its ability to cross the blood-brain barrier in rats (Łabuzek et al., 2010; Lv et al., 2012). Recent studies have shown that metformin acts on the brain (Lv et al., 2012; Kim et al., 2013; Moreira, 2014; Kim et al., 2016; Markowicz-Piasecka et al., 2017), for appetite reduction and the prevention of ischemic brain damage after stroke and dementia. Understanding how metformin affects glucose metabolism when acting on the brain is essential in pharmacological terms to identify drug-organ effects for obtaining a basis for drug development.

We had previously identified that orally administered metformin could activate multiple areas in the brain of obese mice, including hypothalamus, amygdala, nucleus tractus solitarius, and area postrema, thereby decreasing the body weight and food intake (Kim et al., 2016). Notably, the hypothalamus has been found to be directly involved in metformin-induced anorexia (Kim et al., 2013). Therefore, these studies raised the possibility that metformin might regulate peripheral glucose levels by affecting specific brain regions.

The brain, particularly the hypothalamus, regulates energy balance, as well as glucose homeostasis, by sensing nutrients, such as glucose, fatty acids, and amino acids, and integrating signals from the central or peripheral systems including leptin, insulin, and glucagon-like peptide-1 (GLP-1). Glucose homeostasis is mainly regulated via the brain-liver axis, and results in alteration of hepatic glucose production (Lam et al., 2009; Arble and Sandoval, 2013).

Previously, we had revealed that 30 μg of centrally administered metformin induces anorexia via the activation of hypothalamic p70 S6 kinase and reduced the activation of hypothalamic Akt, a key molecule in the insulin signaling pathway (Kim et al., 2013). Hypothalamic S6 kinase not only plays a role in regulating the energy balance, but also participates in peripheral glucose regulation by increasing hepatic glucose production (Cota et al., 2006; Ono et al., 2008; Dagon et al., 2012). Hypothalamic insulin signaling regulates hepatic glucose production via hepatic STAT3 in rodents (Obici et al., 2002; Inoue et al., 2006). Given the beneficial action of metformin on peripheral glucose regulation, we explored whether it influences the peripheral glucose regulatory system via the hypothalamus.

To determine the role of central metformin in glucose regulation, we assessed glucose or insulin tolerance in mice by performing various tests related to glucose regulation after administration of metformin into the third ventricle of mice. We selected a dose of 30 μg based on previous literatures (Stevanovic et al., 2012; Kim et al., 2013). Furthermore, we sought to investigate the mechanism underlying the effects of central metformin administration on peripheral glucose regulation in mice.

## 2 Material and methods

### 2.1 Animals

Seven-week-old male C57BL/6N mice (OrientBio, Seoul, Republic of Korea) were housed individually in a room with controlled humidity and temperature (50%, 22°C ± 2°C), under a 12-h light/dark cycle (light hours, 06:00–18:00). All animals were fed either a chow diet (cat# 5L79, Orient) or 45 kcal % high-fat diet (HFD, cat# d12451, Research Diets Inc., New Brunswick, NJ) *ad libitum*, throughout the experiments. To generate HFD-induced obese group, mice were fed a HFD after their recovery from intra-third ventricle cannulation surgery. The mice were fed a HFD for 10 weeks before glucose tolerance tests.

All procedures followed the guidelines on the ethical use of animals, issued by the Animal Care and Use Committee of Korea University, and were approved by the Institutional Animal Care & Use Committee of Korea University. We have made all efforts to minimize both the number of animals used, as well as animal suffering.

### 2.2 Intra-third ventricle injection

The intra-third ventricle (I3V) cannulation surgery was performed as described previously (Kim et al., 2013). The skull of mice was fixed to a stereotaxic frame (David Kopf Instruments, Tujunga, CA) under anesthesia with ketamine and xylazine (100 mg/kg and 15 mg/kg each). Three screws were inserted around the cannula placement site. After insertion of screws, a 26-gauge guide cannula (Plastics One, Roanoke, VA) was placed into the third ventricle stereotaxically (AP -0.8, DV -4.8 from the bregma). A denture base resin (Vertex RS, Dentimax, Netherlands) was used for fixation. A 30-gauge dummy cannula (Plastics One) was inserted into the guide cannula to maintain patency. All procedures were performed aseptically.

After 1 week of recovery, neuropeptide Y (NPY) challenge test was performed by administering 1 μg/μL of NPY (Sigma-Aldrich, St. Louis, MO) through the cannula at a speed of 2 μL/min to confirm the cannula placement in the 3rd ventricle, as described previously (Kim et al., 2013). Mice that consumed more than 0.5 g of chow diet in 2 h were used for the experiments.

Metformin (1,1-dimethylbiguanide hydrochloride; Sigma-Aldrich) was dissolved in aCSF (artificial cerebrospinal fluid, Tocris Bioscience, Bristol, United Kingdom) at a concentration of 15 μg/μL. Mice were randomly divided into metformin-treated group (MET group) or vehicle-treated group (CON group). There was no difference in body weight between the groups. Two microliters of metformin or vehicle was injected through the cannula at a speed of 2 μL/min, without restraint.

### 2.3 Liver specific sympathetic denervation

Liver specific sympathetic denervation was performed following previous study (Chida et al., 2005). After anesthesia and opening abdominal cavity, portal vein, artery and bile duct were visualized by gently pulling out intestine. 10% (v/v) phenol for liver-specific sympathetic denervation was painted on hepatic artery. After the procedures, peritoneum and abdominal wall were closed with 6–0 polyglycolic acid sutures (Surgifit, AILEE, Seoul, Republic of Korea).

### 2.4 Systemic sympathetic denervation

Mice were intraperitoneally injected with 100 μg/kg of 6-OHDA (6-Hydroxydopamine hydrobromide, Hellobio, Bristol, United Kingdom) on day 1 and 3, and 200 μg/kg of 6-OHDA on day 5. 6-OHDA was dissolved in 0.9% NaCl and 0.1 μM ascorbic acid.

### 2.5 Glucose tolerance test, insulin tolerance test, and pyruvate tolerance test

For glucose tolerance test (GTT), mice were fasted overnight before the experiment. A 20% glucose solution was administered orally (1.5 mg/g) or intraperitoneally (1.5 or 3 mg/g) 30 min after I3V administration of vehicle or metformin. The glucose levels in tail vein blood were measured using a glucometer (Roche Korea, Seoul, Korea) at 15, 30, 60, 120, 180, and 240 min after glucose administration. For pyruvate tolerance test (PTT) and insulin tolerance test (ITT), pyruvate (2 mg/g) or insulin (0.75 U/kg) was administered intraperitoneally according to the body weights. All procedures were performed silently to minimize stress to the animals. All animals were allowed at least 1 week for recovery after each experiment.

### 2.6 Insulin measurement

Serum insulin levels were measured at 0 and 15 min after glucose administration using an Ultra Sensitive Mouse Insulin ELISA Kit (Morinaga Institute of Biological Science, Yokohama, Japan).

### 2.7 Acetaminophen absorption test

A 20% glucose with acetaminophen solution (90 mg/kg, Sigma-Aldrich) was administered intragastrically by oral gavage. Blood was collected from tail vein at 0, 15, 30, and 60 min after oral administration and was stored into heparinized capillary tubes (Kimble chase, Rockwood, TN). Acetaminophen concentration in plasma was measured by acetaminophen diagnostic kits (cat# 505–10, Sekisui Diagnostics, Burlington, MA; cat#ACE4023, Randox, County Antrim, United Kingdom) according to the manufacturer’s instruction.

### 2.8 Quantitative real-time PCR

Two hours after metformin injection, the liver, skeletal muscle, and hypothalamus were harvested, immediately frozen using dry ice, and stored at −80°C. RNA was extracted using TRIzol reagent (Thermo Fisher Scientific, Waltham, MA). cDNA was synthesized using the iScript cDNA Synthesis Kit (Bio-Rad, Hercules, CA) according to the manufacturer’s protocol.

Taqman probes (Thermo Fisher Scientific) were as follows: Glucose 6-phosphatase (Mm00839363_m1), Pck1 (Mm01247058_m1), and Rpl32 (Mm02528467_g1). Gene expression was assessed by real-time quantitative PCR (ABI 7500, Thermo Fisher Scientific). Expression of *Rpl32* was used as an internal control. For statistical analysis, *dCt* values were used and *2* ^*−ddCt*^ values were presented as graphs.

### 2.9 Western blot

Frozen tissues of mice were homogenized in RIPA buffer with phosphatase inhibitors. Western blot was performed as described previously (Kim et al., 2013). Homogenized proteins were quantified using Pierce™ BCA Protein Assay Kit (Thermo scientific, cat#23227). In brief, a total of 20 μg of proteins were separated by 10% SDS-PAGE, and transferred onto PVDF membrane (Bio-Rad). The membrane was incubated overnight with primary antibody, following the manufacturer’s instruction. All antibodies were purchased from Cell Signaling Technology (Danvers, MA); anti-β-Actin antibody (1:4,000, cat# 4,967), anti-STAT3 antibody (1:1,000, cat# 9,132), anti-phospho-STAT3 antibody (1:1,000, cat# 9,131), anti-AMPK antibody (1:2000, cat# 2,532), anti-phospho-AMPK antibody (1:1,000, cat# 2,531), anti-Akt antibody (1:1,000, cat# 9,272), anti-phospho-Akt antibody (1:1,000, cat# 9,275), anti-FoxO1 antibody (1:1,000, cat# 2,880), and anti-IκBα antibody (1:1,000, cat# 9,242) were used. After washing, the membrane was incubated in HRP-conjugated secondary antibody (1:1,000, Cell Signaling Technology) for 2 h at room temperature. Proteins were visualized using an ECL kit (Elpisbiotech, Daejeon, Korea). The intensity of the bands was analyzed using ImageJ software (National Institutes of Health, Bethesda, MD), and normalized to that of an internal control.

### 2.10 Statistical analysis

Normally distributed results are presented as mean ± standard error of the mean (SEM). All analyses were performed using GraphPad Prism 6 software (GraphPad Software Inc., La Jolla, CA). Repeated measures two-way ANOVA was used to assess the significance of differences between groups, time intervals, and interactions in oGTT, ipGTT, ipITT, and ipPTT, followed by Tukey’s multiple comparison tests as the *post hoc* test. We used a Student’s *t*-test on the results of qPCR and western blot. Western blotting results were quantified using ImageJ software. We adopted a significance level of 0.05.

## 3 Results

### 3.1 Central metformin exerts antagonistic effects on glucose tolerance based on the glucose administration route

**FIGURE 1:**
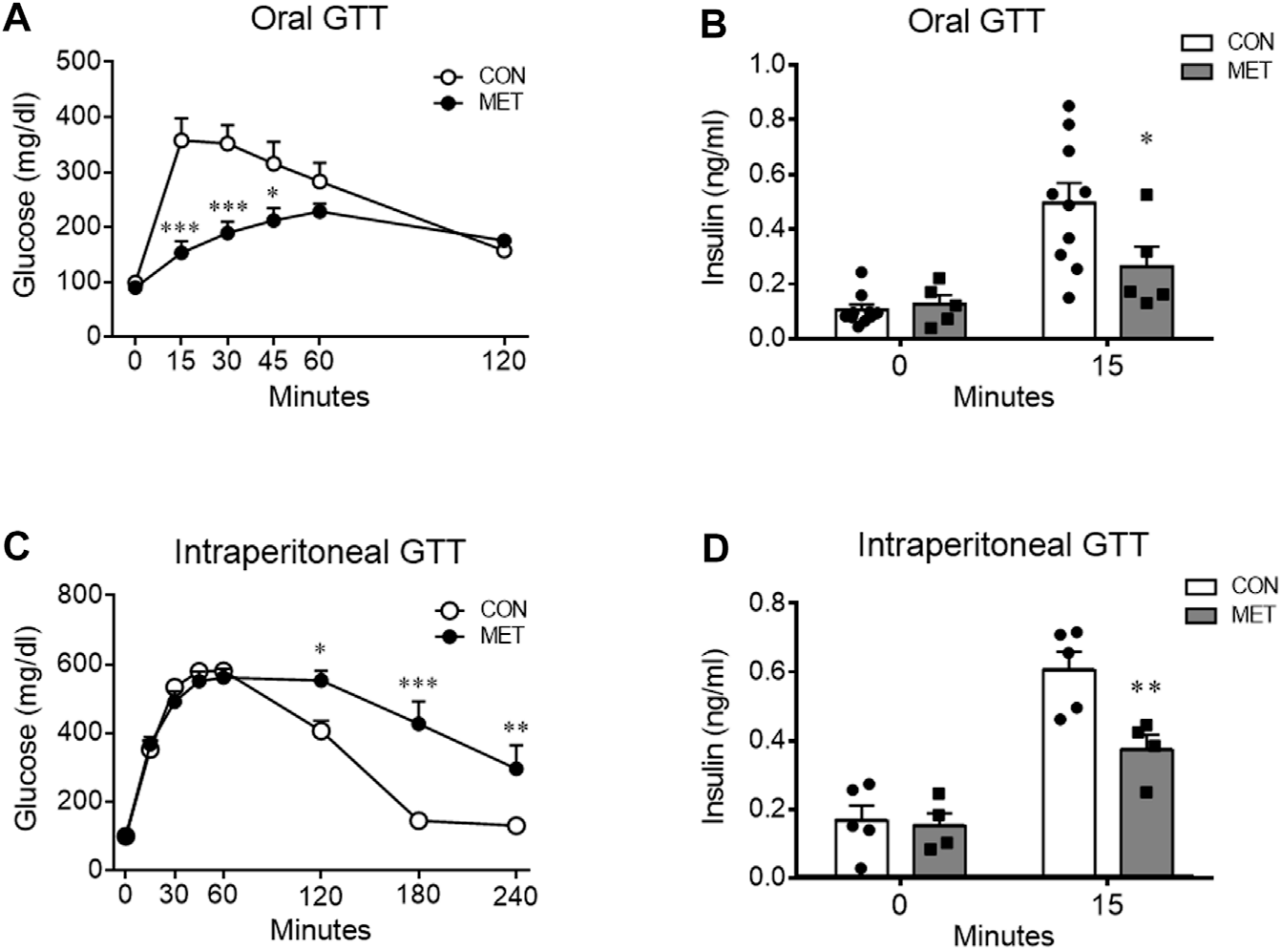
Differential effects of central metformin on glycemic responses based on the route of glucose delivery in mice **(A)** Central effect of metformin on glycemic response to oral glucose **(B)** Comparison of serum insulin levels at 0 and 15 min after the oral glucose load **(C)** Central effect of metformin on glycemic response to intraperitoneal glucose load **(D)** Comparison of serum insulin levels at 0 and 15 min after the intraperitoneal glucose load All glucose tolerance tests (GTT) were performed after 14 h of fasting. Mice were administered glucose orally (1.5 mg/g) or intraperitoneally (3 mg/g) 30 min after delivery of metformin or vehicle into the third ventricle (30 μg).Results are presented as mean ± SEM. CON, group treated with vehicle (*n* = 5); MET, group treated with metformin (*n* = 5); *, *p* < 0.05; **, *p* < 0.01 vs. CON.

To determine whether central metformin regulates peripheral glucose metabolism, we performed glucose tolerance tests via two different routes of glucose administration, namely, intraperitoneal or oral glucose load, after administration of metformin or control into the third ventricle (I3V). We observed a dose-dependent improvement in glucose tolerance in mice that were I3V administered metformin (MET group) compared to those in the control group when glucose was administered orally (Figure 1A; Supplementary Figure S1). In contrast, the glycemic levels significantly increased in the MET group than in the control group when glucose was administered via the intraperitoneal route (Figure 1C).

To investigate the response of insulin secretion on glucose loads, we compared serum insulin levels at 0 and 15 min after oral or intraperitoneal administration of glucose in mice. Two-way ANOVA analysis showed a significant interaction between serum insulin levels across time and treatment (both, *p* < 0.05). The decreased insulin secretion indicated direct suppression by central metformin (Figures 1C, D), considering that there was no significant difference in the 15 min glucose level on ipGTT. Central metformin lowered insulin secretion independently of the glucose administration route in mice (Figures 1B, D).

### 3.2 Central metformin impairs insulin sensitivity by increasing hepatic gluconeogenesis

**FIGURE 2:**
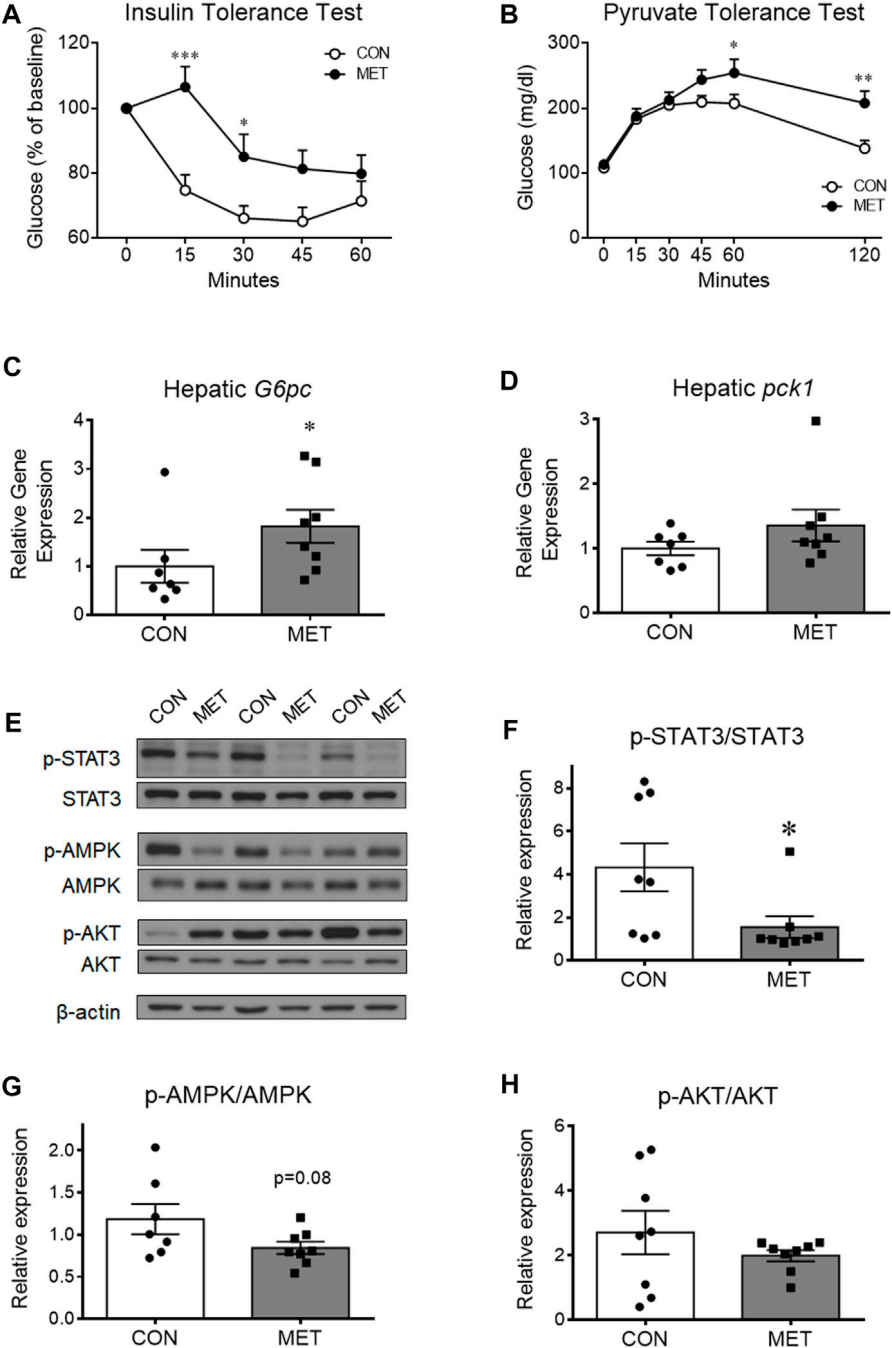
The worsened effect of central metformin on insulin sensitivity and hepatic glucose production in mice **(A)** Central effect of metformin on glycemic response to intraperitoneal insulin load **(B)** Central effect of metformin on glycemic response to intraperitoneal pyruvate load **(C, D)** Comparative expression of hepatic glucose 6-phosphatase (*G6pc*, **(C)** and phosphoenolpyruvate carboxykinase (*Pck1*, **(D)** after delivery of metformin or vehicle into the third ventricle The intraperitoneal insulin and pyruvate tolerance tests were performed after 4 h of fasting. Mice were intraperitoneally administered insulin or pyruvate 30 min after the administration of metformin (30 μg) or vehicle Results are presented as mean ± SEM. CON, group treated with vehicle (*n* = 5); MET, group treated with metformin (*n* = 5); *, *p* < 0.05; **, *p* < 0.01 vs. CON.**(E)** Representative figures showing the levels of phosphorylation of hepatic signal transducer and activator of transcription 3 (STAT3), adenosine monophosphate-activated protein kinase (AMPK), and AKT after delivery of metformin (30 μg) or vehicle into the third ventricle (F, G, and **(H)** Comparison of the levels of phosphorylation of **(F)** hepatic STAT3 (Tyr705), **(G)** AMPK (Thr172), and **(H)** AKT (Thr308) after administration of metformin or vehicle Results are presented as mean ± SEM. CON, group treated with vehicle (*n* = 8); MET, group treated with metformin (*n* = 8); *, *p* < 0.05; **, *p* < 0.01 vs. CON.

To investigate whether central metformin affected the insulin action of lowering blood glucose, we performed an intraperitoneal insulin tolerance test in mice after I3V administration of metformin or control. Glycemic levels were significantly higher in the MET group compared to that in the control group, indicating a decrease in the ability of insulin to lower blood glucose compared to that in control group (*p* < 0.05). This result suggested that central metformin did impair insulin sensitivity in the peripheral tissues (Figure 2A).

To investigate whether central metformin affected hepatic gluconeogenesis, we performed an intraperitoneal pyruvate tolerance test in mice after I3V administration of metformin or control. Orally administered metformin is known to improve insulin sensitivity mainly by decreasing hepatic gluconeogenesis in humans and mice (He et al., 2009; Foretz et al., 2010). However, we found the glycemic levels to be significantly higher in the MET group than in the control group (Figure 2B), indicating that central metformin increased hepatic glucose production compared to that in control. In addition, we found that the expression of glucose 6-phosphatase (*G6Pase*), a gene coding for a rate-limiting enzyme involved in hepatic gluconeogenesis, was significantly higher in the MET group than that in the control group while phosphoenolpyruvate carboxykinase 1 (*Pck1*) expression remained unchanged (Figures 2C, D), consistent with the result from pyruvate tolerance test. Therefore, these results suggest that central metformin decreases insulin sensitivity by enhancing hepatic gluconeogenesis.

### 3.3 Central metformin inactivates hepatic STAT3 signaling

To further investigate the responsiveness of hepatic energy regulators to central metformin, we analyzed and compared the levels of phosphorylated forms of various hepatic energy regulator proteins, such as Akt, 5′ AMP-activated protein kinase (AMPK), signal transducer and activator of transcription 3 (STAT3), IκBα, and FoxO1 after I3V administration of metformin or control (Figures 2E–H; Supplementary Figure S2). We found that STAT3 phosphorylation levels were significantly lower in the MET group compared to that in the control group, whereas AMPK phosphorylation levels tended to decrease (*p* = 0.08) (Figures 2E–G). The levels of total STAT3 and total AMPK (normalized by beta-actin) were not statistically different between groups (Supplementary Figure S3). Given the interconnected action of hepatic STAT3 and the hypothalamus on glucose regulation (Inoue et al., 2006; Kimura et al., 2016), this result suggests that central metformin might worsen glucose tolerance via the brain-liver axis in mice.

### 3.4 Central metformin improves glucose tolerance by delaying gastric emptying

Glucose delivered via oral route passes through the intestine prior to entering the systemic circulation, whereas intraperitoneally administered glucose bypasses the intestine completely. To investigate the mechanism underlying the discrepancy of glucose tolerance between the tests based on different routes of glucose administration, we hypothesized that central metformin might affect peripheral glucose regulation via the intestine.

**FIGURE 3:**
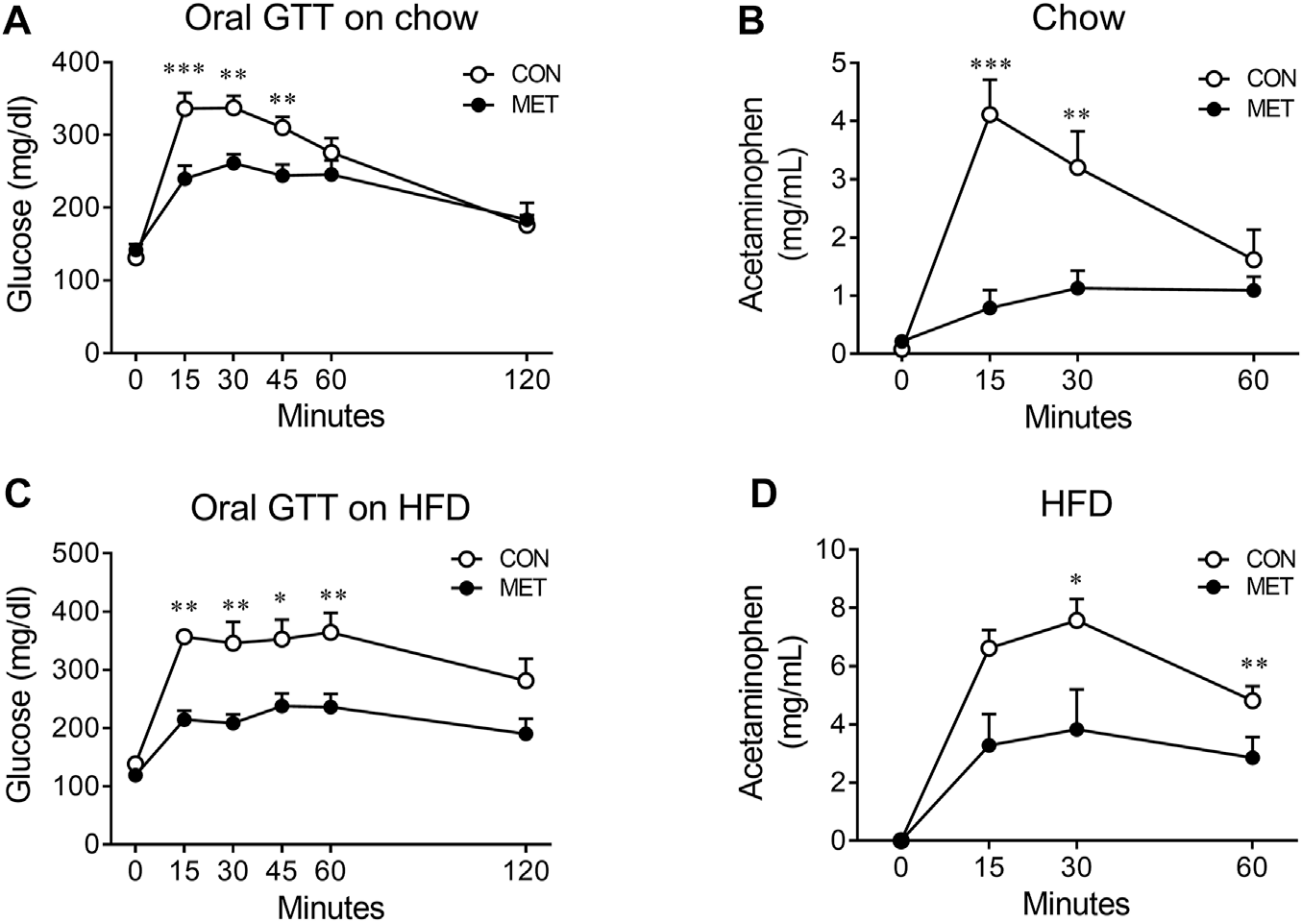
Central effect of metformin on the rate of gastric emptying in mice **(A)** Central effect of metformin on glycemic response to oral glucose in lean mice (*n* = 7, each group) **(B)** Central effect of metformin on gastric emptying in lean mice (*n* = 6, each group) **(C)** Central effect of metformin on glycemic response to oral glucose in high fat diet (HFD)-induced obese mice (*n* = 7, each group) **(D)** Central effect of metformin on gastric emptying in HFD-induced obese mice (*n* = 7, each group) Results are presented as means ± SEM. CON, group treated with vehicle; MET, group treated with metformin (30 μg); *, *p* < 0.05; **, *p* < 0.01 vs. CON.

To test this hypothesis, we compared the rate of gastric emptying in lean mice after I3V administration of either metformin or control, using an acetaminophen absorption test. We found the rate of gastric emptying to be significantly slower in the MET group than in the control group, indicating that the central metformin-induced improvement of glucose tolerance in the oral glucose tolerance test might be attributable to delayed gastric emptying (Figures 3A, B).

To further investigate whether central metformin improves glucose tolerance under conditions of insulin resistance in mice, we checked the glycemic response to an oral glucose load in obese mice fed a high-fat diet for 10 weeks (an average body weight of 39.84 g). We found that central metformin improved glucose tolerance even in obese mice under insulin resistance, compared to control and it also lowered the rate of gastric emptying (Figures 3C, D).

### 3.5 Effect of central metformin on hepatic glucose production is mediated by the sympathetic nervous system

**FIGURE 4:**
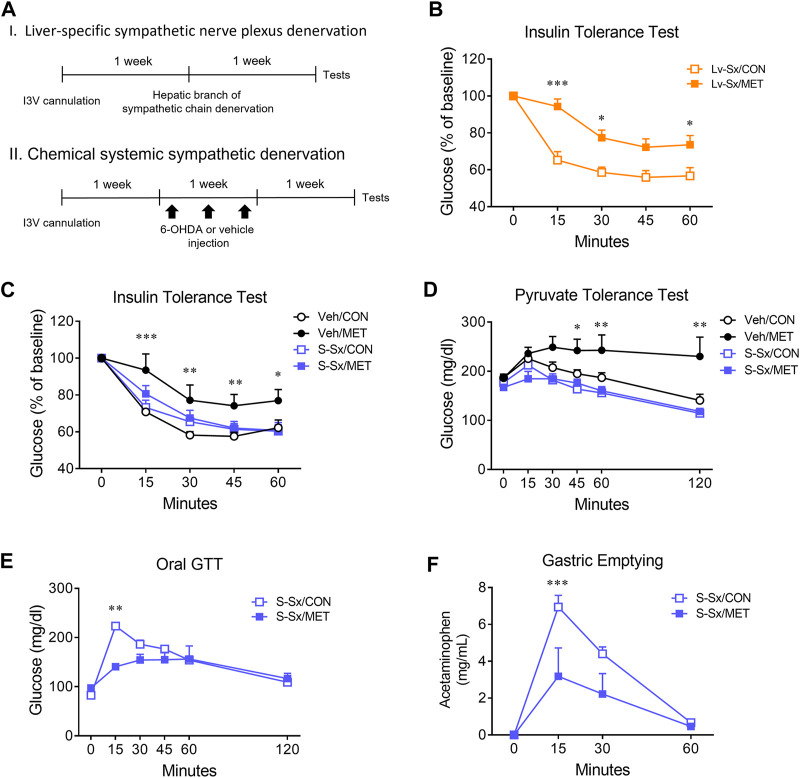
Effect of sympathetic denervation on the central metformin-induced alterations in peripheral glucose regulation **(A)** Experimental diagram of liver-specific sympathetic plexus denervation (I) and systemic sympathetic denervation (II) **(B)** Insulin tolerance test in liver-specific sympathetic denervated mice (Lv-Sx) after icv administration of either metformin or vehicle in mice **(C)** Insulin tolerance test in systemic sympathetic denervated mice (S-Sx) after icv administration of either metformin or vehicle in mice **(D)** Pyruvate tolerance test in systemic sympathetic denervated mice (S-Sx) after icv administration of either metformin or vehicle in mice **(E)** Oral GTT in systemic sympathetic denervated mice (S-Sx) after icv administration of either metformin or vehicle in mice **(F)** Rate of gastric emptying in systemic sympathetic denervated mice (S-Sx) assessed by acetaminophen absorption test during oral GTT after icv administration of either metformin or vehicle in mice Results are presented as means ± SEM. I3V, intra-third ventricle; Lv-Sx, liver-specific sympathetic denervated mice (*n* = 4–8); S-Sx, systemic-specific sympathetic denervated mice (*n* = 4–8); Veh, vehicle-treated mice (*n* = 4–8); *, *p* < 0.05; **, *p* < 0.01; ***, *p* < 0.001 vs. CON.

To investigate the underlying mechanism of the central metformin-induced impairment of peripheral glucose regulation, we performed denervation of the hepatic sympathetic nerve plexus (Lv-Sx) or chemical systemic ablation of the sympathetic nerve (S-Sx) in mice (Figure 4A). Lv-Sx did not change the worsening effect of central metformin on the ability of insulin to regulate glucose levels in mice (Figure 4B), whereas S-Sx suppressed the effects of central metformin on insulin action and hepatic gluconeogenesis (Figures 4C, D). However, central metformin improved glucose tolerance during oral GTT in mice with S-Sx (Figures 4E, F).

## 4 Discussion

**FIGURE 5:**
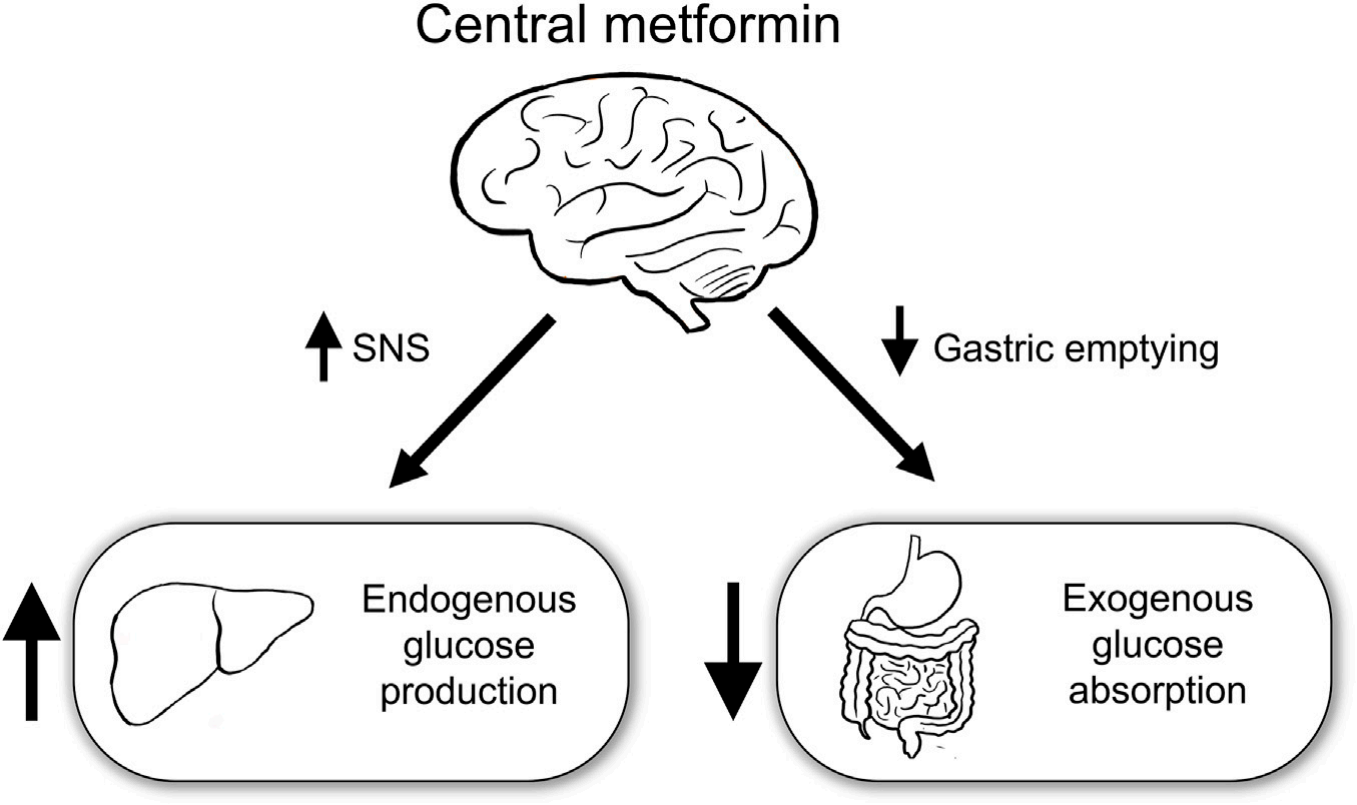
Schematic diagram of the action of central metformin on peripheral glucose regulation Central metformin improves glucose tolerance by delaying gastric emptying via the brain-gut axis and worsens it by concurrently increasing hepatic glucose production via the brain-liver axis. Considering the ordinary way of intake, central metformin may greatly enhance its glucose-lowering action via the brain-gut axis, by surpassing its effect on glucose regulation via the brain-liver axis. SNS, sympathetic nervous system.

This study aimed to identify the role of central metformin in peripheral glucose regulation. Multiple mechanisms underlying the action of metformin in peripheral glucose regulation have already been proposed. However, metformin had also been postulated to regulate peripheral glucose levels via the brain, given its ability to cross the blood-brain barrier and the role of brain in peripheral glucose metabolism (Łabuzek et al., 2010; Arble and Sandoval, 2013). To the best of our knowledge, in this study, we have provided the first evidence that central metformin plays a double-edged role in the regulation of glucose (Figure 5).

Hypothalamus is one of the key regions in the brain that regulates energy balance and participates in glucose regulation. Key metabolic regulators including insulin, leptin, GLP-1, and nutrients (fatty acids, amino acids, and glucose) play a vital role in glucose metabolism via the hypothalamus (Lam et al., 2009; Arble and Sandoval, 2013). In previous studies, we had shown that orally administered metformin activates several brain regions, including the hypothalamus, and metformin produces anorexia and weight loss directly via hypothalamic p70 S6 kinase (Kim et al., 2013; Kim et al., 2016) at a dose of 30 μg. Interestingly, centrally administered metformin also suppressed the activity of hypothalamic Akt, a key insulin signaling molecule (Kim et al., 2013). Given the involvement of hypothalamic p70 S6 kinase and Akt in glucose metabolism, we hypothesized that metformin could regulate glucose metabolism via the hypothalamus (Yue and Lam, 2012).

To test this hypothesis, we investigated the glycemic response to glucose loads via oral or intraperitoneal routes in mice after direct delivery of metformin into the brain. Surprisingly, we observed a discrepancy in the findings of the tests. While centrally administered metformin improved the glycemic response of mice to an oral glucose load compared to that in control, it had an opposite effect to the context of an intraperitoneal glucose load. In a test for analyzing the effect of insulin on glucose metabolism, we found that centrally administered metformin worsened the glycemic response to insulin administration.

Central metformin-induced impairment of glucose metabolism was consistent with the changes in hypothalamic Akt and p70 S6 kinase activities after delivery of metformin into the third ventricle of mice (Kim et al., 2013). Hypothalamic insulin inhibits hepatic glucose production via the brain-liver axis (Schwartz et al., 2013). A high-fat diet causes central insulin resistance, and impairs the ability of insulin to lower hepatic glucose production, which is mediated via activation of hypothalamic p70 S6 kinase (Ono et al., 2008; Muta et al., 2015). Therefore, we investigated whether the central metformin-induced impairment of glucose metabolism could be attributable to increased hepatic glucose production in mice.

Centrally delivered metformin increased the glycemic response to a pyruvate load, compared to that in the control. This was supported by the increased hepatic *G6pc* expression, a rate limiting enzyme of hepatic gluconeogenesis, implying central metformin-induced increase in hepatic glucose production. Hepatic energy regulators, including Akt, AMPK, STAT3, and IκBα, play an important role in peripheral glucose metabolism (Jiang et al., 2008; Hardie et al., 2012). In this study, we observed only a decrease in hepatic STAT3 activity without change in the levels of other. Hepatic STAT3 is known as an effector of the brain insulin-liver interaction that suppresses genes involved in hepatic gluconeogenesis (Inoue et al., 2006). Therefore, our findings strongly support the deteriorated effect of central metformin on glucose metabolism via the brain-liver axis.

It is interesting that metformin, a well-known anti-diabetic agent, worsened glucose metabolism, presumably via the hypothalamus. However, in a test for glucose regulation, when glucose was administered via the oral route, centrally delivered metformin could lower the glycemic levels in mice compared to that in the control, implying that it surpassed the weakened effect of central metformin on glucose metabolism via the brain-liver axis. Its potent effect on peripheral glucose regulation was reproduced in obese mice fed a high-fat diet, which is a typical insulin-resistant animal model (Figures 3C, D). Orally administered glucose is known to enter the body after passing through the small intestine, whereas intraperitoneally administered glucose drains into systemic circulation via the peritoneum, without passing through the intestine. This led us to hypothesize that central metformin may simultaneously exert opposing effects on glucose regulation via the two axis.

The intestine is one of the main organs regulating blood glucose levels (Marathe et al., 2013; Phillips et al., 2015). Peripheral metformin affects intestinal glucose regulation through changes in glucose uptake, GLP-1 secretion, and gut microbiome (McCreight et al., 2016). Based on our results, we assumed that the glucose-lowering action of central metformin might be mediated via the brain-gut axis, likely inhibiting intestinal glucose absorption. The rate of gastric emptying is closely related to postprandial glycemia; even a small decrease in gastric emptying could greatly lower the glycemic levels in humans and rodents (Nicolaus et al., 2011; Marathe et al., 2013; Chambers et al., 2014; Perano et al., 2015). Given that the gastric emptying was much delayed in the group centrally delivered metformin compared to that in the control, the results suggest a central metformin-induced inhibition of intestinal glucose absorption via the brain-gut axis.

The brain regulates peripheral organs primarily through nervous or hormonal signals. When the sympathetic nervous system is activated, hepatic gluconeogenesis increases, but gastric emptying is delayed (Yi et al., 2010; Browning and Travagli, 2014). In this study, systemic sympathetic denervation inhibited central metformin-induced hepatic glucose production, in contrast to liver-specific sympathetic denervation. These results imply the existence of an indirect pathway involving the action of metformin in the brain-liver axis. Central metformin-induced delay in gastric emptying was not mediated by the sympathetic nervous system, suggesting that other pathways may be involved in the mechanism of metformin.

The factors that regulate gastric emptying are unexplored. Factors known till now include characteristics of food consumed (calories, visibility, processing method, total amount, food composition), age, sex, autonomic nervous system (especially the vagal nerve), and gut peptide hormones (Goyal et al., 2019; Liu et al., 2021). The incretin hormones eventually act on the autonomic nervous system to control gastric emptying; we presumed that intracerebroventricular metformin would control gastric emptying through the vagal nerve. To evaluate parasympathetic pathway, we performed a surgical vagotomy. We found that the difference between the groups was not statistically significant (data not shown). The vagal nerve is divided into gastric inhibitory vagal motor circuit (GIVMC) and gastric excitatory vagal motor circuit (GEVMC). They promote and inhibit gastric emptying. The disconnection of both circuits by surgical vagotomy might have contributed to this result and needs further investigation.

This study has several limitations. First, this study was conducted using a mouse model. Metformin injection dose was determined considering previous studies using mouse icv metformin injection model (Sim et al., 2012; Kim et al., 2013; Portela et al., 2015; Brynildsen et al., 2018), but the dose we used in our study may be different with the human oral dose. Further studies are required for its clinical application. Second, we performed only surgical vagotomy. Considering that vagal nerve consists of GIVMC and GEVMC, identifying role of each circuit in metformin’s action is needed. Third, we did not measure serum factors. Some hormones modulate gastrointestinal motility. GLP-1 has been emphasized as a regulator of gastric function. The rate of gastric emptying and secretion of incretin hormones are closely interrelated and form a negative feedback loop in humans (Marathe et al., 2013). In addition, central and peripheral GLP-1 receptors have been proposed to play a role in the regulation of gastric functions in rodents (Imeryüz et al., 1997; Varin et al., 2019). Although the central effect of metformin on GLP-1 secretion in the brain or periphery remains unclear, its action on GLP-1 may contribute to the central metformin-induced delay of gastric emptying via the brain-gut axis, which lowers intestine glucose absorption. Further studies are needed to investigate the involvement of GLP-1 in the central metformin-induced changes in peripheral glucose metabolism.

## 5 Conclusion

In this study, we proposed that metformin acts directly in the brain on peripheral glucose regulation in mice. Central metformin was shown to have a dual effect on the glycemic response depending on the route of glucose administration. We suggest that central metformin may suppress exogenous glucose absorption by delaying gastric emptying via the brain-gut axis, and it may worsen endogenous glucose metabolism by increasing the hepatic glucose production via the brain-liver axis. Metformin is one of the most useful antidiabetic agents; understanding its mechanisms would expand its usage or aid the development of better strategies for treating diabetes. Therefore, this study may be a forerunner to guide the development of new strategies to potentiate the effects of metformin on glucose regulation.

## Data Availability

The original contributions presented in the study are included in the article/Supplementary Material, further inquiries can be directed to the corresponding author.
