# Supplementary material for: Metformin acts as a dual glucose regulator in mouse brain
Source: Front Pharmacol. 2023 Apr 20;14:1108660. doi: 10.3389/fphar.2023.1108660 (PMC10157063; doi:10.3389/fphar.2023.1108660)
Supplement: Supplementary file 1 [file Table1.DOCX]

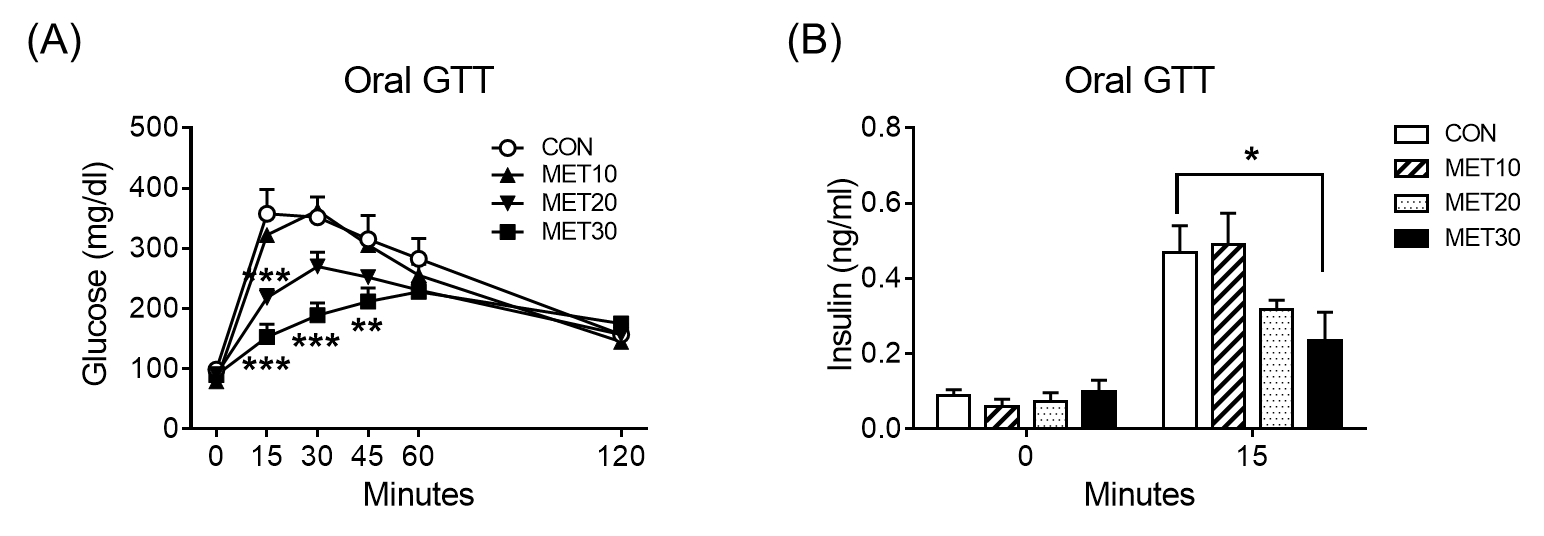


**Supplementary figure 1. oGTT result after various doses of central metformin administration**

(A) Oral glucose tolerance test and

(B) Plasma insulin concentration at 0 and 15 min after I3V metformin administration

Results are presented as mean ± SEM. CON, group treated with vehicle (n = 5); MET10, group treated with 10 μg of metformin (n = 5); MET20, group treated with 20 μg of metformin (n = 5); MET30, group treated with 30 μg of metformin (n = 5). **, *p* < 0.01; ***, *p* < 0.001 vs. CON.

**
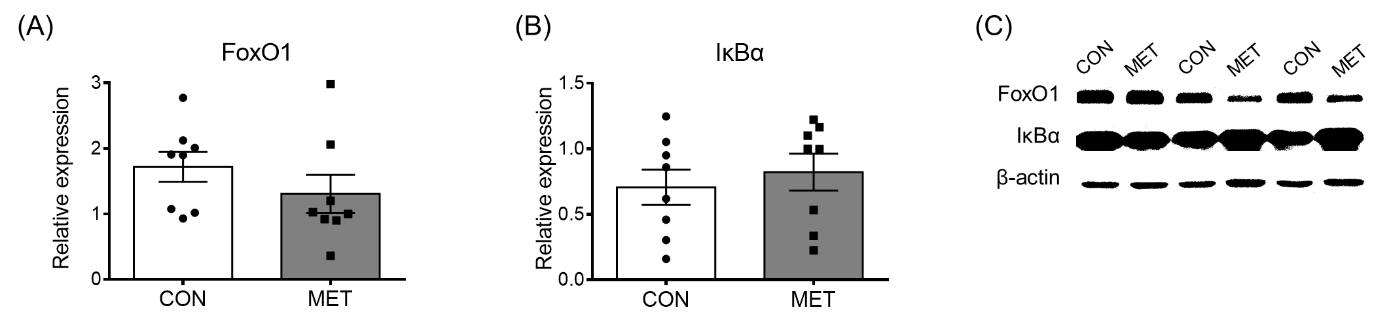
**

**Supplementary figure 2. Central effect of metformin on the activities of hepatic energy regulators in mice**

Comparison of the levels of phosphorylation of hepatic (A,C) IκBα, and (B,C) FoxO1 after delivery of metformin (30 μg) or vehicle into the third ventricle.

Results are presented as means ± SEM. CON, group treated with vehicle (n = 8); MET, group treated with metformin (n = 8); *, *p* < 0.05; **, *p* < 0.01 vs. CON.


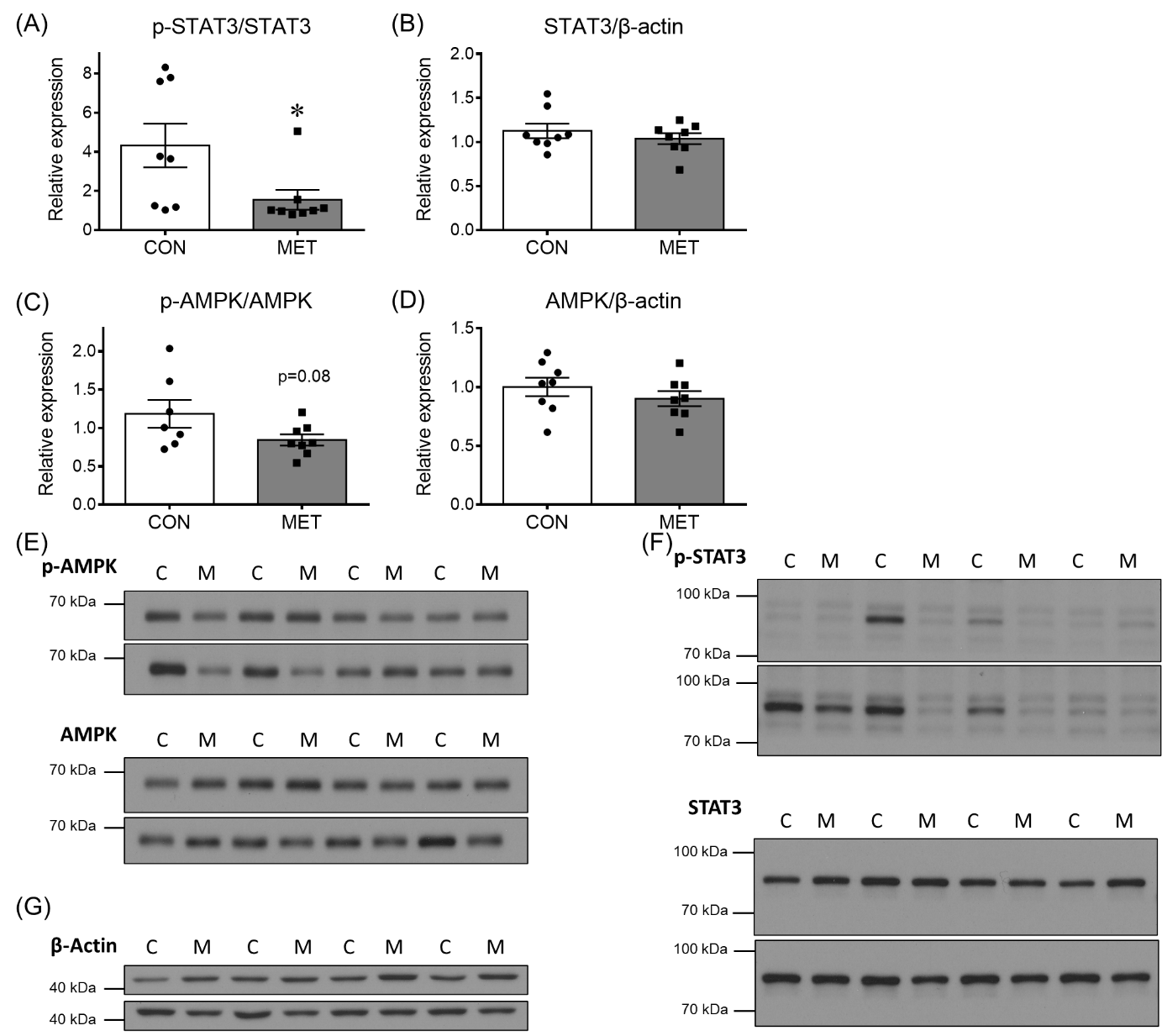


**Supplementary figure 3 Protein expression in liver**

Comparison of the levels of phosphorylation of hepatic (A,F) STAT3, (C,E) AMPK and total protein level of (B) STAT3, (D) AMPK normalized with (G) beta actin after delivery of metformin (30 μg) or vehicle into the third ventricle.

Results are presented as means ± SEM. CON/C, group treated with vehicle (n = 8); MET/M, group treated with metformin (n = 8); *, *p* < 0.05; **, *p* < 0.01 vs. CON.


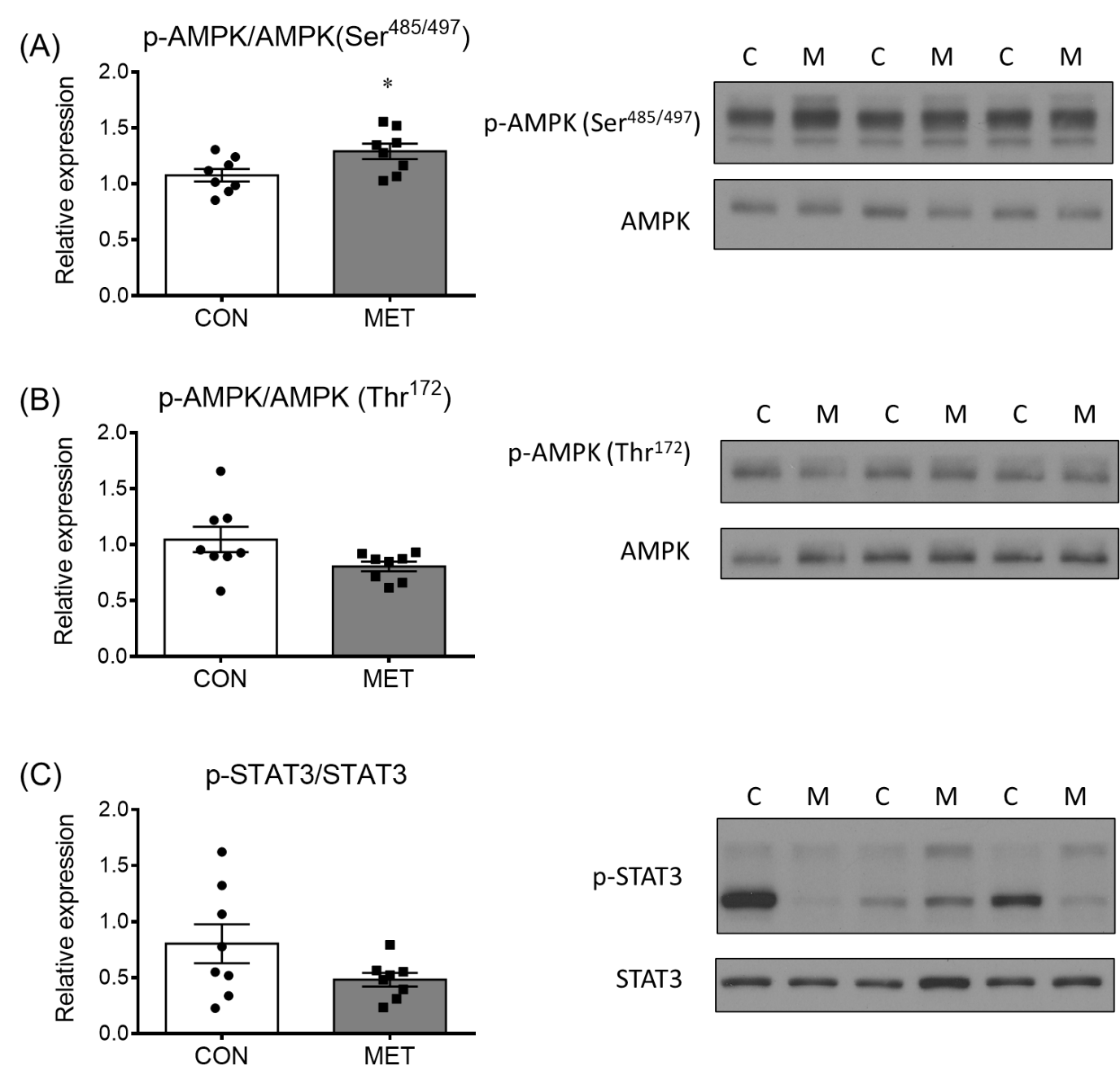


**Supplementary figure 3 Protein expression in hypothalamus**

Comparison of the levels of phosphorylation of hypothalamic (A) AMPK (Ser485/497) (B) AMPK (C) STAT3 after delivery of metformin (30 μg) or vehicle into the third ventricle.

Results are presented as means ± SEM. CON/C, group treated with vehicle (n = 8); MET/M, group treated with metformin (n = 8); *, *p* < 0.05; **, *p* < 0.01 vs. CON.
